# Last but not least: emerging roles of the autophagy-related protein ATG4D

**DOI:** 10.1080/15548627.2024.2369436

**Published:** 2024-06-26

**Authors:** Emily McMann, Sharon M. Gorski

**Affiliations:** a Canada’s Michael Smith Genome Sciences Centre at BC Cancer, Vancouver, British Columbia, Canada; b Department of Molecular Biology and Biochemistry, Simon Fraser University, Burnaby, British Columbia, Canada; c Centre for Cell Biology, Development and Disease, Simon Fraser University, Burnaby, British Columbia, Canada

**Keywords:** Atg8, cancer, GABARAPL1, mitochondria, neurodegeneration, neurodevelopmental disorders

## Abstract

The evolutionarily conserved ATG4 cysteine proteases regulate macroautophagy/autophagy through the priming and deconjugation of the Atg8-family proteins. In mammals there are four ATG4 family members (ATG4A, ATG4B, ATG4C, ATG4D) but ATG4D has been relatively understudied. Heightened interest in ATG4D has been stimulated by recent links to human disease. Notably, genetic variations in human *ATG4D* were implicated in a heritable neurodevelopmental disorder. Genetic analyses in dogs, along with loss-of-function zebrafish and mouse models, further support a neuroprotective role for ATG4D. Here we discuss the evidence connecting ATG4D to neurological diseases and other pathologies and summarize its roles in both autophagy-dependent and autophagy-independent cellular processes.

**Abbrevation**: ATG: autophagy related; BafA1: bafilomycin A1; BCL2: BCL2 apoptosis regulator; BH3: BCL2 homology region 3; CASP3: caspase 3; EV: extracellular vesicle; GABA: gamma aminobutyric acid; GABARAP: GABA type A receptor-associated protein; GABARAPL1: GABA type A receptor associated protein like 1; GABARAPL2: GABA type A receptor associated protein like 2; GFP: green fluorescent protein; LIR: LC3-interacting region; MAP1LC3: microtubule associated protein 1 light chain 3; MEF: mouse embryonic fibroblast; MYC: MYC proto-oncogene, bHLH transcription factor; PE: phosphatidylethanolamine; PS: phosphatidylserine; QKO: quadruple knockout; SDS-PAGE: sodium dodecyl sulfate-polyacrylamide gel; SQSTM1: sequestosome 1.

## Introduction

**Figure 1.**
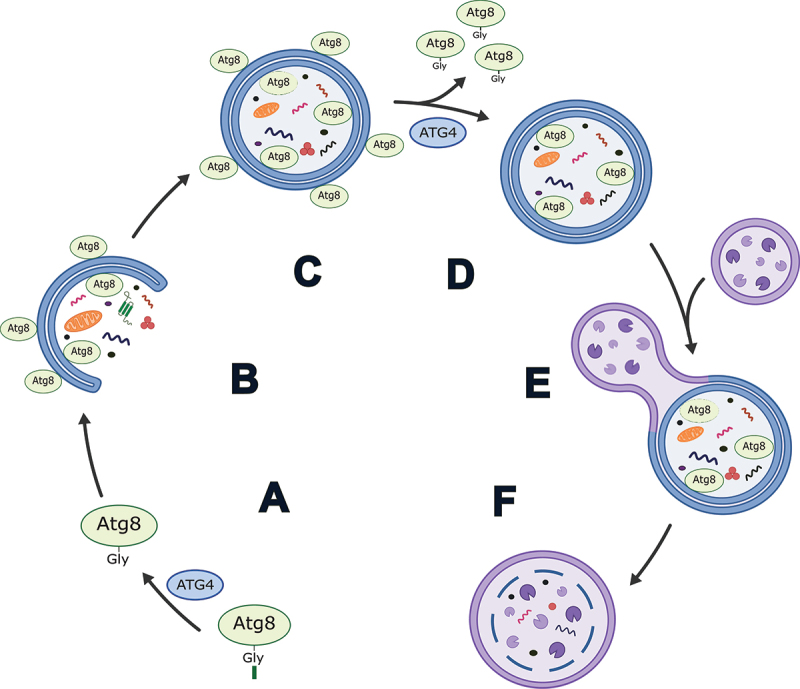
Simplified schematic of the role of ATG4 in autophagy in mammals. (A) Priming of an Atg8-family protein via cleavage after the C-terminal glycine by ATG4. (B) Conjugation of Atg8-family proteins to the developing phagophore membrane. (C) Autophagosome formation. (D) Delipidation of Atg8-family proteins from the outer autophagosome membrane by ATG4. Note: exact timing of this process has yet to be determined in mammals, but it is thought to occur prior to and/or shortly after autophagosome-lysosome fusion. (E) Autophagosome-lysosome fusion. (F) Autolysosome formation and degradation of cargo.

Autophagy is a highly conserved intracellular recycling process that serves to maintain cellular homeostasis and promote stress adaptation. Phagophores engulf cellular components such as proteins, organelles, and macromolecular complexes, and mature into double membrane bound structures termed autophagosomes, which then fuse with lysosomes to form autolysosomes (Figure 1). This fusion event exposes autophagosomal cargo to the hydrolytic enzymes of the lysosome, resulting in cargo breakdown and the subsequent release of the building blocks (amino-acids, sugars or lipids) of the degraded components back into the cytoplasm for reuse. Autophagy occurs at basal levels in all eukaryotic cells but can be upregulated in response to stresses such as starvation, oxidative damage, or the accumulation of protein aggregates, among others [1,2]. Consequently, autophagy dysregulation has been associated with aging and various pathologies such as neurodegeneration and cancer [3,4].

The formation of the autophagosome is regulated by more than 30 ATG (autophagy related) proteins which function in the initiation, expansion and maturation of the phagophore, and fusion of the autophagosome with the lysosome [5,6]. Among these proteins are the ATG4 cysteine proteases and ubiquitin-like Atg8-family proteins. In yeast, there is a single Atg4 and Atg8, both of which are required for autophagy [7]. Atg4 cleaves pro-Atg8 to form Atg8-I, exposing a key glycine residue at the C terminus. This step is referred to as “priming” and allows for the conjugation of Atg8 to phosphatidylethanolamine (PE) in the phagophore membrane to produce lipidated Atg8 (Atg8-II), a step necessary for expansion and closure of the membrane [7–11]. Atg4 is also responsible for the subsequent delipidation/deconjugation of Atg8 from the outer autophagosome membrane prior to or shortly after vacuole/lysosome fusion (Figure 1) [11–18].

In humans there are four ATG4 homologs (ATG4A, ATG4B, ATG4C, ATG4D) that share a conserved C54 endopeptidase domain, but possess divergent N- and C -termini. Further, there are seven human Atg8-family proteins, divided into two subcategories: the MAP1LC3/LC3 (microtubule associated protein 1 light chain 3 proteins, consisting of LC3A, LC3B, LC3B2, and LC3C), and the GABARAP (GABA type A receptor-associated protein) subfamily, which consists of GABARAP, GABARAPL1 (GABA type A receptor associated protein like 1), and GABARAPL2 [19]. In mice, LC3B2 and LC3C do not exist, but the other ATG4 and Atg8-family members are conserved. The four human ATG4 homologs possess varying affinities and processing capabilities toward the Atg8-family members [20–22]. However, ATG4B is thought to be the main priming ATG4 in mammals [22–24]. In contrast, ATG4D possesses only weak priming ability toward Atg8-family proteins, although cleavage of ATG4D by CASP3 (caspase 3) increases its priming and delipidation activities *in vitro* [21,22,25]. Mammalian cells with *ATG4D* knockout are viable but loss-of-function mutations, knockout, and knockdown of *ATG4D in vivo* leads to neurodegeneration in mice, canines, fish, and humans [26–29]. These discoveries suggest ATG4D may have an important role to play in maintaining cellular homeostasis, but it remains unclear whether these phenotypes are the result of a defect in autophagy and/or other cellular processes.

## ATG4D and Atg8-family members

### Macroautophagy

ATG4D has been reported to possess very low *in vitro* priming activity for all tested Atg8-family proteins and was shown to be the least active of all the ATG4 homologs (ATG4B>ATG4A>ATG4C>ATG4D) in conditions utilizing soluble Atg8-family protein substrates. However, subsequent investigations, detailed below, demonstrated that ATG4D cleavage activity is affected by its post-translational processing and the lipidation state of Atg8-family substrates [20–22,25]. Li et al. [22] performed an *in vitro* assessment of the activity of recombinantly expressed ATG4D against soluble LC3B, GABARAP, GABARAPL1, and GABARAPL2 with C-terminal GST (glutathione S-transferase) fusions, where successful cleavage of the Atg8-family fusion proteins was determined by the accumulation of free GST during SDS-PAGE analysis. In these conditions, ATG4D shows no activity against GABARAP and GABARAPL1 and very low activity toward GABARAPL2 and LC3B that is comparable to a catalytic mutant of ATG4B (ATG4B^C74S^), despite having a similar GABARAPL2 affinity (*1/K*_*m*_) as ATG4B. Betin and Lane [21] also assessed the *in vitro* priming capability of ATG4D for LC3 (specific subfamily member not indicated), GABARAPL1, and GABARAPL2 by assessing changes in the molecular weight of C-terminally MYC (MYC proto-oncogene, bHLH transcription factor)-tagged proteins during western blot analysis. In agreement with Li et al. [22], ATG4D is ineffective at priming all these Atg8-family proteins. However, *in vitro* priming assays with recombinantly expressed protein may not accurately represent the substrate range of ATG4D as it does not account for the potential post-translation modifications, protein interactions, and effects of lipid-anchored substrates present *in vivo*. ATG4B undergoes various post-translational modifications [30–32], and the remaining ATG4 homologs may also be regulated by post-translational modification events. Notably, ATG4D can be cleaved by CASP3 at the cleavage site DEVD_63_K to generate ΔN63 ATG4D [21]. Accordingly, GABARAPL1 and weak GABARAPL2 priming are detected by *in vitro* experiments with the N-terminally truncated ΔN63 ATG4D. These results indicate that the N terminus of ATG4D may have an autoinhibitory effect as its removal increases proteolytic activity, and that ΔN63 ATG4D is likely the active form of ATG4D in cells [21,33,34].

Mammalian cell line studies provide further insight into the substrate range of ATG4D. In *ATG4A ATG4B ATG4C* triple-knockout HeLa cells, lipidated GABARAPL1 and lipidated GABARAPL2 are still detectable, which indicates that these proteins have undergone priming by ATG4D [20]. Surprisingly, in these cells GABARAPL1 and GABARAPL2 lipidation are unaffected by *CASP3* knockdown, pointing toward potential priming activity of full-length ATG4D. However, it is possible any residual CASP3 is sufficient for the production of ΔN63 ATG4D, and evaluation for the presence of this fragment during *CASP3* knockdown was not performed. Additionally, the possibility of other unknown post-translational modifications to ATG4D that affect its priming capabilities in triple-knockout cells cannot be ruled out. In a separate study, protein levels of six Atg8-family members were tested in *ATG4A ATG4B ATG4C ATG4D* quadruple knockout (QKO) HeLa cells expressing mCherry-ATG4D [35]. Only GABARAPL1 is cleaved under these conditions, although mCherry-ATG4D does appear to have a stabilizing effect on pro-GABARAP levels. Morimoto et al. [29] similarly report the presence of primed GABARAPL1 in *ATG4* QKO HeLa cells expressing full-length ATG4D. However, in *ATG4* QKO HEK293 cells stably overexpressing ATG4D or ΔN63 ATG4D, only trace amounts of lipidated GABARAPL1 can be detected along with significant amounts of unprimed GABARAPL1, suggesting only minimal priming activity from ATG4D or ΔN63 ATG4D [25]. Finally, loss of ATG4D in otherwise wild-type cells does not appear to lead to obvious priming defects, as *atg4d* knockout mouse embryonic fibroblasts (MEFs) and *ATG4D* knockdown HeLa cells display no deficiency in the priming of pro-Atg8-family proteins, although HeLa cells do have reduced green fluorescent protein (GFP)-tagged GABARAPL1-positive autophagosomes [21,26]. These observations suggest that the activity of the remaining ATG4 homologs is sufficient for the priming of Atg8-family members. While ATG4D is capable of priming some Atg8-family members, with a preference for GABARAPL1 as a priming substrate, the cellular contexts of ATG4D-mediated priming of Atg8-family proteins remain unclear.

In addition to priming, ATG4s are also responsible for delipidating Atg8-family members from PE in autophagosomal membranes. Delipidation occurs at much lower rates than the initial priming step, which may function to prevent the premature removal of Atg8-family members from the forming autophagosomes [25]. In yeast, recognition and delipidation of PE conjugated Atg8 by Atg4 is reliant on two conserved amino acid motifs: an N-terminal Atg8–PE association region/APEAR and a C-terminal LC3-interacting region (LIR) [18,36]. Human ATG4B contains an evolutionarily conserved Atg8–PE association region [36], and both human ATG4B and ATG4D possess a C-terminal LIR [25]. In ATG4B, this LIR determines lipidated Atg8-family interactions and therefore may serve a similar function in ATG4D [25]. Along with improved priming abilities, ΔN63 ATG4D also has increased delipidation activities compared to full-length ATG4D. Kauffman et al. [25] incubated full-length ATG4D and ΔN63 ATG4D with synthetic liposomes containing LC3B, GABARAPL1, and GABRAPL2 conjugated to PE. Lipidated Atg8-family proteins migrate faster during gel electrophoresis than their unlipidated counterparts, and liposomes incubated with full-length ATG4D do not show conversion of any of the tested Atg8-family proteins to their slower migrating delipidated forms when analyzed by SDS-PAGE. However, ΔN63 ATG4D demonstrates delipidation activity against all three tested Atg8-family proteins (also analyzed by SDS-PAGE), with an apparent preference for LC3B. In a similar experiment, full-length ATG4D does not demonstrate delipidation activity when incubated with autophagosome-enriched membrane fractions from cells stably expressing yellow fluorescent protein-tagged LC3 or GFP-GABARAPL1, but ΔN63 ATG4D can delipidate GFP-GABARAPL1 [21]. Cell line and animal models with alterations in ATG4D levels support an active role for ATG4D in delipidation. Loss of *atg4d* in murine cells results in defects in the removal of membrane-bound Atg8-family members (primarily LC3B) from the cytosolic leaflet of autophagosomal and autolysosomal membranes, which is not seen in *atg4a*, *atg4b*, or *atg4c* null mutants. This phenotype is enhanced through the additional loss of ATG4C in *atg4c atg4d* double-knockout MEFs [26,37]. Consistent results are seen in human HCT116 *ATG4D*^*-/-*^ cells that accumulate lipidated LC3B, GABARAPL1, and GABARAPL2 [38]. Furthermore, *atg4d*^*-/-*^ mice have increased levels of lipidated LC3A, LC3B, GABARAP, GABARAPL1, and GABARAPL2 [26]. Again, this phenotype is enhanced through the additional loss of ATG4C in *atg4c atg4d* double-knockout mice [37]. Collectively, these observations indicate that ATG4D may function primarily in Atg8-family member delipidation, possibly as the main delipidating ATG4 in mammals [26], although other studies point to significant contribution from ATG4C [37] or to the equal contribution of all ATG4s to delipidation [25,39].

In yeast, Atg8 delipidation is important for autophagosome formation as it provides a pool of primed Atg8 while also removing inappropriately lipidated Atg8 proteins that accumulate on various organelle membranes within the cell [11,12,16]. The exact role or timing of delipidation in relation to autophagy in mammalian cells remains unclear, but there is evidence to suggest that the delipidation activities of ATG4 homologs prevent the aberrant conjugation of Atg8-family proteins to various non-autophagosomal intracellular membranes [35], although this function has not been specifically demonstrated for ATG4D [20,26,35]. While loss of ATG4D is associated with an increase in lipidated Atg8-family members, this accumulation is not necessarily detrimental to autophagy function, as the expression of a pre-primed variant of GFP-LC3B can rescue autophagy in *ATG4* QKO HeLa cells, indicating autophagy can proceed in the absence of ATG4-mediated delipidation [20]. Additionally, increased levels of LC3B can be found on the cytosolic leaflet of autolysosome membranes in *atg4d*^*-/-*^ MEFs, suggesting autophagosomes lacking LC3B delipidation are still capable of fusing with lysosomes [26]. Further, a N-terminally hemagglutinin/HA-tagged pre-primed variant of GABARAPL1 can be removed from the outer membrane of autophagosomes and autolysosomes in HeLa cells lacking all ATG4s [35]. Regardless, some loss-of-function mutations in *ATG4D* still possess phenotypes that could point toward a defect in autophagosome-lysosome fusion, such as the accumulation of autophagosomes observed in uterine fibroids with reduced *ATG4D* expression and *atg4d*^*-/-*^ MEFs [26,40]. While not necessary for autophagosome-lysosome fusion, delipidation may increase the efficiency of autophagy. However, it is important to remember that Atg8-family members have additional cellular roles independent of autophagy [41,42]. Alterations in the dynamics between cytosolic primed and lipidated Atg8-family proteins could affect other processes unrelated to autophagy, such as the conjugation of Atg8-family members to other proteins within the cell (a process known as Atg8ylation) [35,43]. ATG4s have also been implicated in Atg8ylation through the removal of Atg8-family members from Atg8ylated proteins, although this specific function has not been demonstrated for ATG4D [35].

### ATG4D and endocytic membranes

Atg8-family members can be conjugated to phosphatidylserine (PS) in single-membrane endolysosomal compartments produced from various endocytic engulfment events, such as phagocytosis, micropinocytosis, and entosis [39,44]. In addition to the deconjugating ability of ΔN63 ATG4D for PE-lipidated Atg8-family proteins during autophagy, full-length ATG4D is also capable of delipidating LC3B and GABARAP from PS and PE in single membranes [38,39,45,46]. Furthermore, ATG4D is more efficient at delipidating LC3B – PS than ATG4B from liposomes containing both LC3B – PS and LC3B – PE *in vitro*, where relative presence of each conjugate was determined by mass spectrometry [39]. In cells, *ATG4D* knockout leads to the accumulation of LC3B – PS, although the remaining Atg8-family members have yet to be tested in this context [39]. Therefore, loss of ATG4D may lead to alterations in autophagy-independent engulfment events.

## ATG4D in and around the mitochondria

**Figure 2.**
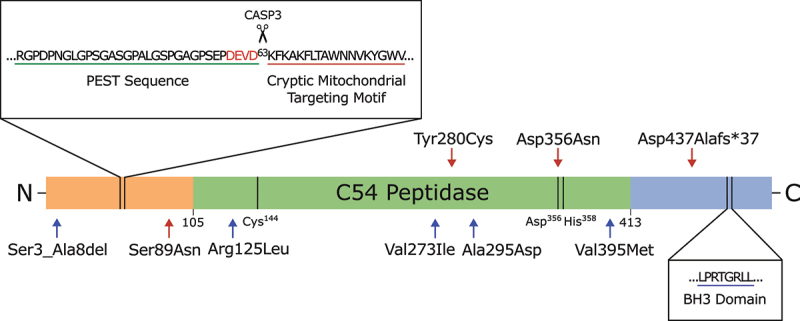
Linear representation of human ATG4D. The 474 amino acid long protein consists of a conserved C54 peptidase domain flanked by N- and C-termini that are divergent from the other three ATG4 homologs. The N terminus contains a CASP3 recognition site (DEVD) that can be cleaved to produce ΔN63 ATG4D. Immediately upstream of this cleavage site is a putative PEST sequence, which suggests ATG4D is a short-lived protein subject to degradation by the proteasome. Immediately downstream is a cryptic mitochondrial motif that is exposed upon CASP3 cleavage. The C terminus contains a putative BH3 domain and LIR motif [21,22,25]. Red arrows indicate known mutations in human ATG4D that are associated with neurodegeneration [29] and blue arrows indicate mutations potentially associated with non-obstructive azoospermia [17].

Immediately downstream of the N-terminal DEVD_63_K site in human ATG4D is a cryptic mitochondrial targeting sequence (Figure 2), and exposure of this sequence via CASP3 cleavage results in the localization of ΔN63 ATG4D to the outside of the mitochondria and the mitochondrial matrix [47]. In the mitochondrial matrix ΔN63 ATG4D is subject to further processing by mitochondrial proteases, resulting in ~ 42-kDa and ~ 45-kDa products which likely correspond to ATG4D with the N terminus plus mitochondrial targeting motif removed and an intermediate product between ΔN63 ATG4D (~47 kDa) and the ~ 42-kDa form, respectively. However, CASP3 cleavage is apparently unnecessary for ATG4D import into the mitochondria as a caspase-resistant mutant of ATG4D (ATG4D^D63A^) is still recruited to the mitochondria, and the ~ 42-kDa form of ATG4D can always be detected in the mitochondria, even in the presence of the pan-caspase inhibitor zVAD.fmk [21,47]. Further evidence for a role of ATG4D in the mitochondria comes from the affinity of ΔN63 ATG4D to liposomes containing 20 mol percent of the mitochondrial-specific lipid, cardiolipin. This affinity is not possessed by any of the remaining ATG4 homologs [25]. As noted by Betin et al. [47], it is unlikely that ATG4D is accessing Atg8-family members while residing in the mitochondria. However, the role of ATG4D in the mitochondria may still involve its endopeptidase activity because the overexpression of ΔN63 ATG4D-GFP, but not the endopeptidase active site mutant ΔN63 ATG4D^C144A^-GFP, leads to a significant reduction of mitochondrial cristae density [47]. While it is unlikely ATG4D is performing its canonical role of Atg8-family member priming and delipidation from within the mitochondrion, it is possible that its presence within this organelle is still related to autophagy. Oxidative stress induced via H_2_O_2_ exposure can cause ATG4D to associate with the mitochondria [21], indicating that ATG4D may be responding to perceived mitochondrial damage and is attempting to clear damaged mitochondria by initiating mitophagy [47,48]. In addition to responding to oxidative stress, ATG4D may also be an instigator of this damage through an as-of-yet-unknown pathway. ATG4D associated reduction in mitochondrial cristae density may decrease the efficiency of oxidative phosphorylation, thereby leading to an increase in the production of reactive oxygen species/ROS [47,49,50]. Accordingly, human erythroid cells expressing ΔN63 ATG4D-GFP show an increase in MitoSOX fluorescence and therefore production of mitochondrial reactive oxygen species [47]. However, in *Drosophila melanogaster*, overexpression of Atg4b (hereafter “DmAtg4b”), the fly ortholog of human ATG4D, is protective against H_2_O_2_ induced oxidative stress [51]. In the mitochondrion, ATG4D may be instigating its own recruitment, perhaps to initiate ATG4D driven clearance through mitophagy. Consistent with a role in mitophagy, ATG4D is necessary for the clearance of mitochondria during erythropoiesis [52], and reintroduction of ATG4D in *ATG4* QKO HeLa cells is able to restore mitophagy, even more efficiently than the reintroduction of ATG4B [35]. Additionally, GABARAPL1, a known substrate of ATG4D, is involved in mitophagy [19,53].

The observation that ATG4D is subject to cleavage by CASP3, a protease that typically functions as a pro-apoptotic effector, suggests that ATG4D could also be involved in the cell death process. The cleavage of ATG4D to ΔN63 ATG4D exposes a putative BCL2 homology region 3 (BH3) domain which potentially binds BCL2 (BCL2 apoptosis regulator)-family members present on the mitochondria, thereby inducing mitochondrial outer membrane permeabilization. Accordingly, the overexpression of ATG4D and ΔN63 ATG4D in HeLa cells is cytotoxic, and the transient association of these proteins with the outside of the mitochondria is associated with apoptosis and loss of mitochondrial membrane potential [21]. Further, deletion of the BH3 domain in GFP-ΔN63 ATG4D significantly reduces the cell death associated with the overexpression of this construct in HeLa cells [21]. Interestingly, loss of ATG4D through siRNA-mediated knockdown in pancreatic ductal adenocarcinoma cell lines HPAC and SW1990 also leads to loss of mitochondrial potential, but ANXA5-PI staining revealed apoptosis is reduced in these cells [48].

## Pathologies associated with alterations in ATG4D

### Canine ATG4D and neurodegenerative vacuolar storage disease

ATG4D plays an important role in neuron homeostasis, and mutations in ATG4D have been implicated in heritable neurodegenerative diseases in humans and canines [26,27,29] (Table 1). Kyöstilä et al. [27] describe the first link between ATG4D and an inherited neurodegenerative disease in the Lagotto Romagnolo breed of dog. This autosomal recessive condition, which they term neurodegenerative vacuolar storage disease/NVSD, is the result of a single nucleotide change (G1288A) in the last exon of *ATG4D*, the outcome of which is a predicted missense mutation where an alanine is replaced by a threonine residue (ATG4D^A430T^). Dogs homozygous for this allele display various progressive neurological phenotypes including behavioral changes, mild atrophy of the cerebellum and forebrain, cerebellar ataxia, and loss of Purkinje and granular cells. Another striking feature associated with neurodegenerative vacuolar storage disease is extensive cytoplasmic vacuolization in cells of the nervous system, secretory tissues, epithelia, and of mesenchymal origin. The vacuoles are bound by a single membrane and differ in size between tissue types, with many appearing to contain little to no sequestered material. The identity of these vacuoles has yet to be determined, but they possess markers for lysosomes, autophagosomes, and endosomes, implicating the involvement of the endosomal and autophagy machinery in their formation [54–57]. Fibroblasts isolated from affected dogs and cultured under basal conditions show an increase in immunofluorescent LC3 puncta compared to controls, and western blot analyses performed on the same cells show an increase in the lipidated forms of LC3 proteins. Upon treatment of the variant fibroblast cells with bafilomycin A_1_ (BafA1), the differences in levels of lipidated LC3 proteins between control and ATG4D variant cells disappear, suggesting the accumulation of lipidated LC3 proteins in the untreated ATG4D variant cells may be the result of a blockage in autophagosome-lysosome fusion [54]. Of note, no differences in LC3 proteins between control and ATG4D variant cells are observed under starvation conditions, suggesting only basal autophagy is altered. Alternatively, these results could reflect alterations of autophagy-independent but LC3-associated processes [58–61], such as LC3-dependent extracellular vesicle loading and secretion/LDELS [62]. ATG4D-variant canine fibroblasts show an increase in the release of extracellular vesicles (EVs) [57]. As with lipidated LC3 levels, this difference disappears during starvation. The release of these EVs from variant fibroblasts may be dependent on LC3-associated processes, and therefore increased levels of lipidated LC3 May be directly associated with the increased release of EVs under basal conditions rather than from an increase in LC3 associated with autophagosomes.

**Table 1.** Summary of neurodegenerative phenotypes associated with knockdown, knockout, or loss-of-function mutations of various *ATG4D* orthologs.

Species	ATG4D ortholog	Alteration/manipulation	Notable phenotypes	Reference
Canines (*Canis lupus familiaris*)	*ATG4D*	Predicted loss-of-function mutation	Behavioral changes, progressive cerebellar ataxia and loss of Purkinje cells, accumulation of cytoplasmic vacuoles, increased EV release, altered basal autophagy	[27,54,57]
Zebrafish (*Danio rerio*)	*Atg4da*	Knockdown	Malformed CNS, Purkinje cell loss, neurodegeneration, hydrocephalus	[27]
Mice (*Mus musculus*)	*Atg4d*	Knockout	Loss of Purkinje cells, cerebellar ataxia, motor coordination defects, accumulation of autophagosomes, altered basal autophagy, mislocalization of GABA_A_ receptors	[26]
Human (*Homo sapiens*)	*ATG4D*	Loss-of-function mutation	Motor coordination defects, cerebellar hypoplasia, various neurological symptoms (e.g., seizures)	[26,29]

EVs from ATG4D-variant cells have a more diverse protein content than controls, with a relative increase in proteins from the cytosol, mitochondria, and the endoplasmic reticulum, and a relative decrease in proteins derived from the nucleus, lysosome, and cytoskeleton [57]. EV proteomes from ATG4D-variant cells also contain relatively more extracellular matrix proteins and proteins with chaperone activity. Interestingly, the autophagic cargo marker SQSTM1 (sequestosome 1) is present in EVs from ATG4D-variant fibroblasts, but not controls, and this may point to a rerouting of undegraded cargo to EVs from autophagosomes that are unable to fuse with lysosomes. Interestingly, inhibition of autophagosome-lysosome fusion through *ATG7* knockout and BafA1 treatment or via the administration of chloroquine enhances the accumulation of autophagy-related proteins, including SQSTM1, inside EVs in cell lines and mouse models [62,63]. While autophagy is traditionally viewed as a digestive pathway, its machinery has also been implicated in secretion via EV release [62–65]. It is possible that mutations in ATG4D could directly affect EV loading and/or production through alterations in processes such as LC3-dependent extracellular vesicle loading and secretion, or could indirectly affect EV production by increasing cellular levels of undegraded materials that require rerouting to EVs, and these areas warrant further research.

### ATG4D in zebrafish and mice

The function of ATG4D has also been explored in zebrafish and mice, and knockdown or knockout of ATG4D in these models leads to neurological phenotypes consistent with those observed in canines [26–28] (Table 1). Knockdown of *atg4da* (a functional homolog of ATG4D) in zebrafish embryos leads to severe malformations in the developing central nervous system, as well as Purkinje and granular cell depletion and widespread neurodegeneration. *atg4d*^−*/-*^ mice demonstrate a significant reduction in the numbers of Purkinje cells in the cerebellum, cerebellar ataxia, overall reduced neurological function, and motor coordination defects that progress with age. These phenotypes are all consistent with the role of Purkinje cells in motor control and cognition [66]. The extensive vacuolization of tissues observed in canines is not conserved in zebrafish and mice, although the accumulation of small autophagosomes can be seen in mice [26]. Like canines, *atg4d*^*-/-*^ mice tissues show an accumulation of membrane bound Atg8-family proteins, GFP-LC3B puncta, and SQSTM1. However, unlike canines, these phenotypes, with the exception of SQSTM1 accumulation, are also present during starvation conditions. Increases in membrane bound Atg8-family proteins are also observed in *atg4d*^*-/-*^ (but not *atg4a, atg4b*, or *atg4c* knockout) MEFs. It is likely that the increase in accumulation of membrane-bound Atg8-family proteins is due to reduced delipidation activity in the absence of ATG4D.

Closer examination of Purkinje cells in mice reveals ultrastructural features consistent with dark cell degeneration [26], a type of cell death associated with excitotoxicity and mitochondria dysfunction [67,68]. GABARAP interacts with the ionotropic inhibitory GABR (gamma-aminobutyric acid type A receptor) and plays a part in its trafficking to the plasma membrane [69]. *atg4d*^−*/-*^ neurons show an increase in this GABR-GABARAP interaction, as well as reduced localization of GABR to the plasma membrane and increased targeting of GABR for lysosomal degradation. Since alterations in ATG4D have been shown to cause defects in the levels of lipidated Atg8-family proteins, it was hypothesized that mutations in ATG4D contribute to neurodegeneration by causing an accumulation of PE-conjugated GABARAP, and potentially also PE-conjugated GABARAPL1 and GABARAPL2 [70,71]. The increase in GABARAP – PE could in turn alter GABR localization and reduce inhibitory signals in Purkinje cells, leading to subsequent excitotoxicity and cell death through an autophagy-independent pathway. Extraordinarily, the administration of 4,5,6,7-tetrahydroisoxazolo[5,4-c]pyridin-3-ol (THIP), a GABR agonist, rectifies the motor coordination defects associated with *atg4d* knockout when administered to young mice, perhaps by enhancing inhibitory signals to Purkinje cells and thereby preventing excitotoxicity [26]. This hypothesis links the degeneration of Purkinje cells to a defect in Atg8-family protein processing. Autophagy may still prove to play a role in this Purkinje cell degeneration, as parallels exist between phenotypes seen in *atg4d* deletion and *atg5* and *atg7* deletion mice [72,73], but it is also possible there are other unknown implications of ATG4D dysfunction. Interestingly, specific overexpression in the nervous system of the Drosophila ortholog of ATG4D, DmAtg4b, has been linked to increased lifespan, further suggesting a neuroprotective role for ATG4D [51]. Further investigation is warranted regarding the implications of ATG4D loss-of-function on mitochondria in Purkinje cells, as ATG4D has been found to localize to mitochondria, and both ATG4D and the mitochondrion have been implicated in dark cell degeneration [47,68].

### ATG4D alterations in humans are associated with a neurodevelopmental disorder

**Figure 3.**
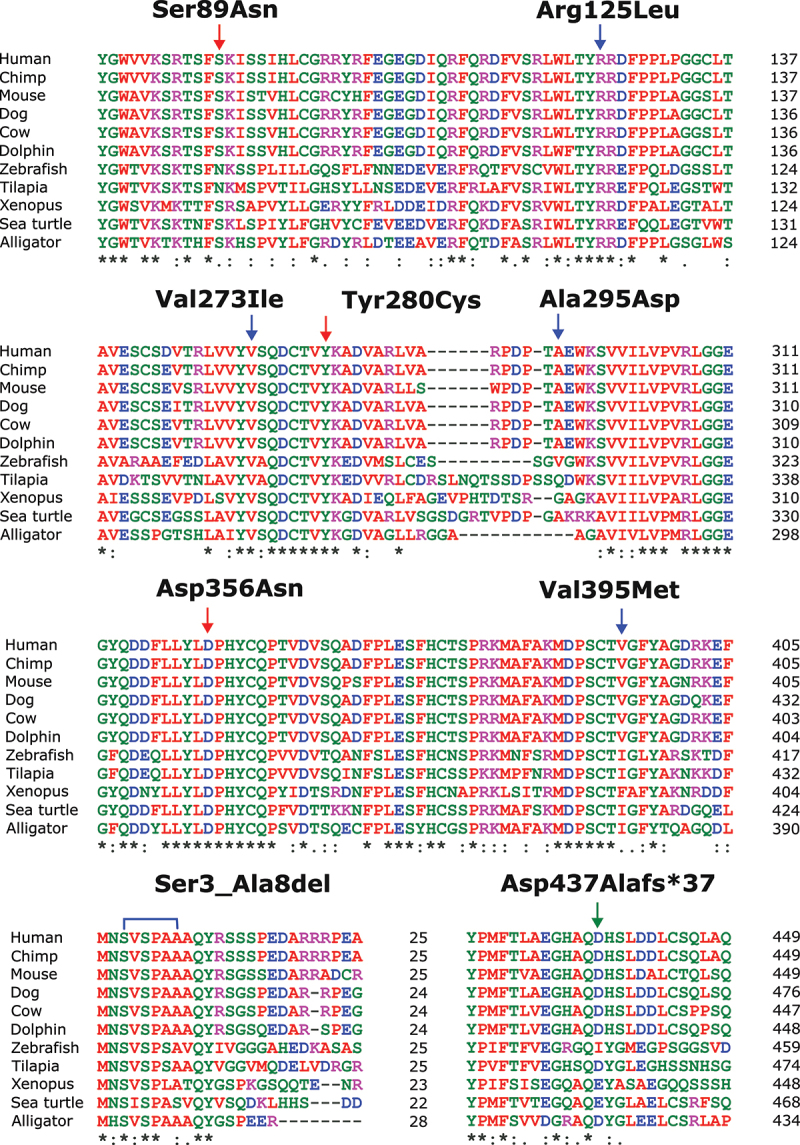
Conservation of residues Ser89, Arg125, Val273, Tyr280, Ala295 Asp356, and Val395 across various vertebrate species. Red arrows indicate site of amino acid change, and green arrow indicates the beginning of a deletion site and frameshift in variant human ATG4D proteins associated with a neurodevelopmental disorder [29]. Blue arrows indicate site of amino acid changes and blue square brackets indicate deleted region in variant human ATG4D proteins associated with non-obstructive azoospermia, as reported by the authors [17]. Asterisks “*” indicate identical amino acids, colons “:” indicate conserved amino acids, and periods “.” indicate semi conserved amino acids. Alignments performed using ClustalW [74]. NCBI accession numbers: Human (Homo sapiens), NP_116274.3; Chimp (Pan troglodytes), XP_016790485.1; Mouse (Mus musculus), NP_705811.8; Dog (Canis lupus familiaris), XP_038284569.1; Cow (Bos taurus), NP_001092616.1; Common bottlenose dolphin (Tursiops truncates), XP_033710226.1; Zebrafish (Danio rerio), XP_009292712.1; Nile tilapia (Oreochromis niloticus), XP_003447971.1; Xenopus (Xenopus tropicalis), NP_001039246.1; Green sea turtle (Chelonia mydas), XP_037743907.1; American alligator (Alligator mississippiensis), XP_006265658.2.

ATG4D has been implicated in a rare autosomal recessive syndromic neurodevelopmental disorder in humans. Tamargo-Gómez et al. [26] first describe a compound heterozygous individual possessing two variants of *ATG4D* with missense mutations (ATG4D^S89N^ and ATG4D^Y280C^) and apparent loss of function. Morimoto et al. [29] have described two additional compound heterozygous individuals (siblings) with a missense mutation and a deletion that leads to a frameshift in *ATG4D* (ATG4D^D356N^ and ATG4D D437Afs *37, respectively). Notably, D356N directly disrupts the catalytic triad of ATG4D (Figure 2). Individuals with these variants present with varying severity of symptoms and disease progression, with phenotypes reminiscent of those seen in mice and canines, such as abnormal gait, cognitive impairment, and mild cerebellar atrophy. Primary fibroblast and lymphoblastoid cell lines from affected individuals do not demonstrate consistent alterations in lipidated LC3 or GABARAP family proteins compared to controls under basal conditions or upon BafA1 treatment, so these mutations may not alter autophagy induction or autophagic flux, at least under these conditions. To test the GABARAPL1 priming capabilities of these variants, N-terminally cleaved ATG4D variant proteins were incubated with GABARAPL1-MYC *in vitro*. Cleavage was assessed via western blot using the shift in molecular weight of GABARAPL1 upon removal of the MYC tag. It was found that all variants except ATG4D^S89N^ have reduced GABARAPL1 priming activity [29]. Interestingly, asparagine is present at the analogous site to Ser89 in some ATG4D orthologs (Figure 3), and this may explain the observed lack of effect on GABARAPL1 priming *in vitro*. Additionally, in *ATG4* QKO HeLa cells, ATG4D^Y280C^ and ATG4D^D356N^ are unable to completely rescue GABARAPL1 priming [29], although when expressed in ATG4D-deficient MEFs, ATG4D^Y280C^ and ATG4D^S89N^ are able to partially rescue the accumulation of lipidated Atg8-family proteins [26]. Given that previous evidence indicates a more important role for ATG4D in delipidation rather than priming, it is worth further investigating the effect of these mutations on the processing of lipidated Atg8-family proteins. Further, since the phenotypes associated with the ATG4D variants are of a neurodegenerative nature, only so much insight can be gained by the study of non-neuronal cells, and ATG4D may have tissue specific roles that can only be brought to light through the study of neuronal cells.

### ATG4D and male infertility

Five recessive mutations in *ATG4D* have also been potentially associated with male infertility in humans via non-obstructive azoospermia (Figure 2) [17]. These mutations were predicted to be pathogenic by in silico analyses and were detected in four patients: two siblings with a homozygous variant and two unrelated patients with heterozygous variants. Germ cells from the two patients with homozygous *ATG4D* variants were reported to show decreased levels of ATG4D and LC3B as determined by immunofluorescence, as well as an increase in TUNEL-positive cells, though quantification was not provided. ATG4D is highly expressed in the testes (Human Protein Atlas, proteinatlas.org) [75] and has been shown to be a target of the AR (androgen receptor) transcription factor [76], but its precise role in germ cells and spermatogenesis has yet to be elucidated. Interestingly, males homozygous or compound heterozygous for the *ATG4D* variants do not display any neurodegenerative phenotypes and are otherwise healthy.

### ATG4D and cancer

Autophagy can play a dual role in the development and progression of cancer. Initially, it can act to suppress tumorigenesis by managing stressors such as oxidative damage through the removal and degradation of damaged organelles. However, after tumor cells have become established, autophagy can contribute to their survival in low nutrient and hypoxic conditions [77,78]. Of the four human *ATG4* homologs, *ATG4D* is the most frequently altered in cancers [40,79]. These alterations in *ATG4D*, which can include changes in methylation, increases and decreases in transcript and protein levels, as well as alterations in the protein itself, are not consistent between various cancer types (Table 2) [79,87]. These differences may be reflective of the dual roles of autophagy in cancer, and/or to tissue-specific differences in roles and regulation of ATG4D, and/or may alternatively reflect passenger alterations in *ATG4D* in some cancer contexts. For example, reduced expression of *ATG4D* has been observed in uterine fibroids and may contribute to their development [40]. Uterine fibroid cell lines (HuLM) and tissues exhibit increased numbers of autophagosomes, but have significantly fewer autolysosomes, suggesting there may be a defect in the fusion of autophagosomes and lysosomes. Increases in LC3-II and LC3-I are observed in these cells, and this may indicate defects in delipidation as well as the conjugation of LC3-I to PE. Importantly, knockdown of ATG4D in normal myometrial cell lines promotes the proliferation and survival of these cells, producing a phenocopy of uterine fibroid cells. Therefore, reduced *ATG4D* expression may be a driver in the formation of uterine fibroids, perhaps by reducing effective autophagic flux and its cytoprotective effect. On the other end of the spectrum, Zhao et al. [86] showed *ATG4D* expression is significantly increased in hepatocellular carcinoma tissues. *In vitro* assays with MHCC-97 L cells revealed that silencing *ATG4D* inhibited cell proliferation, promoted apoptosis, and decreased AKT phosphorylation while simultaneously increasing CASP3 levels, so ATG4D may possibly act on the AKT-CASP3 pathway. Likewise, overexpression of *ATG4D* in IMR-90 cells decreases the levels of SQSTM1 and of senescence-associated proteins CDKN1A (cyclin dependent kinase inhibitor 1A) and CDKN2A, suggesting increased levels of ATG4D promote autophagy and cell proliferation [89]. In this context, ATG4D and autophagy may be promoting progression in an already established tumor.

**Table 2.** Altered expression of *ATG4D* and its associated protein in various cancer and tumor types.

Tissue/Cancer type	Stage	mRNA expression levels	Protein expression levels	Reference
Acute myeloid leukemia	G1	Increased (RNA-Seq)	-	[80]
Acute myeloid leukemia	-	Decreased (RTqPCR)	-	[81]
Cervical cancer	-	Decreased (RTqPCR)	-	[82]
Colorectal tumors	-	Decreased (RTqPCR)	-	[83]
Gastric cancer	III-IV	Decreased (microarray)	-	[84]
Glioma	-	Increased (RTqPCR)	Increased (WB)	[85]
Head and neck squamous cell carcinoma	G4	Increased (RNA-Seq)	-	[80]
Hepatocellular carcinoma	-	Increased (RTqPCR)	Increased (WB)	[86]
Invasive ductal carcinomas of the breast	-	Decreased (RTqPCR)	-	[87]
Lung squamous cell carcinoma	-	Increased (RNA-Seq)	-	[80]
Uterine fibroids	-	Decreased (RTqPCR)	Decreased (WB, IF)	[40]
Uterine fibroids	-	-	Decreased (WB)	[88]

Method for determination of mRNA and protein expression in brackets. IF: immunofluorescence; WB: western blot; RTqPCR: reverse transcription quantitative polymerase chain reaction; RNA-Seq: RNA-sequencing. (-) indicates not reported or not determined.

**Figure 4.**
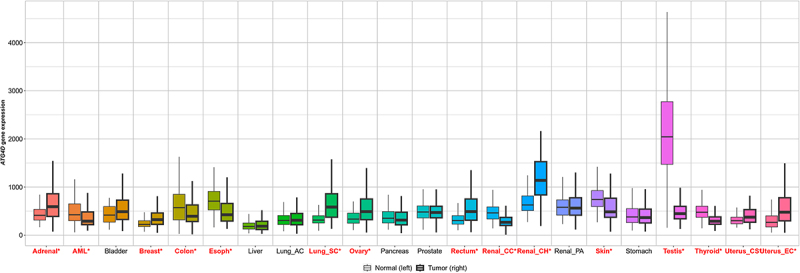
Pan-cancer analysis of ATG4D expression in normal (left, non-bold) and tumor (right, bold) tissues as determined by RNA-Seq. Tissue types marked in red demonstrate significant differences in ATG4D expression (Mann-Whitney U test, *p < 0.01). Analysis and figure generated using TNMplot [94]. AC: adenocarcinoma; AML: acute myeloid leukemia; CC: clear cell carcinoma; CH: chromophobe; CS: carcinosarcoma; EC: corpus endometrial carcinoma; Esoph: esophagus; PA: papillary cell carcinoma; SC: squamous cell carcinoma.

*ATG4D* is also a target of the tumor suppressing microRNA, *MIR101*. *MIR101* can reduce levels of *ATG4D* mRNA, which supresses autophagy, promotes apoptosis, and sensitizes some cancer types to chemotherapy treatments [79,90–93]. The observed variability in ATG4D-related effects in various cancer contexts may also be due in part to the different genetic backgrounds of the cell line models used for the studies. A pan-cancer analysis using RNA-Seq demonstrates the variability of *ATG4D* expression across various tissue and tumor types (Figure 4) [94]. Further investigations are needed to delineate the roles of ATG4D in cancer progression and treatment response. Despite its apparent role in cancer development and progression, increased levels of *ATG4D* expression have also been associated with prolonged lifespan in humans [89] and Drosophila [51].

## Concluding remarks and future perspectives

One of four ATG4 family members in mammals, ATG4D is emerging as an important player in Atg8-family member delipidation and in human disease. In addition to the delipidation of double-membrane bound Atg8-family member proteins in canonical autophagy, ATG4D has been implicated in the delipidation of Atg8-family proteins from single-membrane organelles. Hence, the roles of ATG4D in regulating conjugation of Atg8 to single membranes (CASM), specifically in the conjugation and delipidation of Atg8-family proteins to PE or PS, requires further study. The relationship of ATG4D to extracellular vesicle formation, loading and release also remains to be investigated. A related question is how these processes are affected by CASP3-mediated cleavage of ATG4D, which is shown to generate ΔN63 ATG4D and to increase ATG4D catalytic activity. The upstream regulation of this ATG4D cleavage event, its *in vivo* contexts, effects on ATG4D subcellular localization, and whether other proteases can similarly process ATG4D are important areas for future investigation. Additional modes of ATG4D post-translational modification, and potential non-catalytic roles, also remain to be elucidated. Importantly, how do all of these molecular events affect normal physiology and disease pathogenesis? Multiple lines of evidence from several different species now support a role for ATG4D in neuroprotection. Does ATG4D have distinct roles or interaction partners in neuronal versus non-neuronal cells? The discovery of the first *ATG4D* mutations associated with a human neurodevelopmental disorder raises many questions that warrant attention. Could ATG4D alterations be associated with a spectrum of neurodevelopmental disorders and are there existing cases still waiting to be discovered? Is ATG4D-related mitochondrial dysfunction involved? What molecular, cellular and physiological processes are affected by these *ATG4D* allelic variants, and can we use such information to devise effective therapeutic strategies? While studies of ATG4D may have lagged in comparison to some of the other ATG4 family members, these important questions highlight the need to propel forward ATG4D-related investigations in both normal development and disease contexts.

## Data Availability

The data used to generate Figure 4 are available in TNMplot at http://tnmplot.com [94].
